# Supplementary material for: Are carbon emissions trading and green financial instruments synergistic? -Comprehensive quantitative research based on content analysis
Source: PLoS One. 2024 Mar 7;19(3):e0298601. doi: 10.1371/journal.pone.0298601 (PMC10919601; doi:10.1371/journal.pone.0298601)
Supplement: S1 Data — (ZIP) [file pone.0298601.s001.zip › Data/地级市Z_F工作报告.zip!/╕╩╦α/└╝╓▌╩╨/└┤╫╘╣1⁄2╓┌║┼í╛┬φ┐╦╩2╛▌═°í┐_2012.docx]

各位代表：

　　现在，我代表市人民政府向大会作工作报告，请予审议，并请各位政协委员和其他列席人员提出意见建议。

　　一、五年政府工作回顾

　　即将过去的五年，是兰州经济社会发展最快、城乡面貌变化最大、城市影响力快速提升、人民生活持续改善的五年。五年来，在省委、省政府和市委的正确领导下，在市人大、市政协的监督支持下，市政府团结和带领全市各族人民，以科学发展观为统领，全面贯彻国办支持甘肃发展《若干意见》和省委区域发展战略，认真落实市委“1355”总体发展思路和“再造兰州”战略，积极应对各种困难和挑战，集中力量破解发展难题，顺利完成了“十一五”规划和市十四届人大一次会议确定的各项目标任务，全市经济社会发展迈上新的台阶。

　　一是综合经济实力显著增强。全市主要经济指标连续保持两位数增长，年均增速高于全国和全省平均水平，全部实现翻番增长。预计今年生产总值将达到1360亿元，是2006年的2.13倍。全社会固定资产投资可达950亿元，是3.19倍。社会消费品零售总额可达638亿元，是2.2倍。地区性财政收入和地方财政总收入可达350亿元和150亿元，分别是3.3倍和3.6倍。建成了兰州石化大乙烯、榆中钢厂、西固热电联产等大型产业支撑项目，规模以上工业增加值可达460亿元，是2.17倍。非公有制经济增加值由194亿元增加到544亿元，是2.8倍，占生产总值的比重由30%提高到40%。单位生产总值能耗、污染减排等约束性指标全面完成。

　　二是破解发展难题实现突破。针对发展空间受限难题，立足发挥中心带动作用，实施“再造兰州”战略，启动兰州新区建设和两个国家级开发区增容扩区，城市规划区面积由1649平方公里扩大到5810平方公里，市区规划面积由221平方公里扩大到688平方公里。针对产业支撑不足难题，积极研究发展培育多元支柱产业，确定了石油化工、有色冶金、装备制造、能源和新能源、战略性新兴产业、现代服务、文化旅游、特色农产品深加工等重点产业，编制了全市园区和产业布局规划，制定了“研政产”体系等一系列政策措施。针对环境污染难题，开展大气污染成因及防治对策研究，制定实施环境污染治理行动计划，深入推进大气和水污染治理，城市大气污染综合指数下降17%，饮用水及黄河兰州段水质达标率保持100%，生活污水处理率达到95%，兰州被列为全国大气污染治理试点城市。针对交通拥堵难题，大力推进“畅交通”工程，坚持远近结合、建管并重、标本兼治，做到集中整治、强化管理和完善路网相结合，城市交通拥堵问题正在逐步解决。

　　三是城乡环境面貌明显改善。在全力支持中央和省属公路、铁路、机场等基础设施建设的同时，坚持高起点规划、高标准建设，五年累计投入600多亿元，实施了雁滩南河道、南北滨河路延伸、南山路、黄河大桥、污水“全收集全处理”等重大项目。推动小街巷、城中村、棚户区、国有企业危旧房和城市出入口“五大改造”，整治小街巷700多条，完成61个城中村“无形改造”，建成村民安置房100万平方米，改造棚户区和国有企业危旧房450万平方米。加大城市环境综合整治，推进城市绿化、亮化、净化和美化，全市森林覆盖率达到12.21%，主城区绿地率达到26.7%。城乡一体化试点工作扎实推进，小城镇和新农村建设取得新进展，城镇化率由59%提高到76%。加强农村水、电、路、气、房等基础设施建设，实施大型泵站更新改造，新建改造农村公路5200公里，解决54万农民饮水安全问题，建成农村户用沼气池4万多座，减少农村贫困人口5.28万人。

　　四是改革开放步伐不断加快。全面实施国有企业改革“393”攻坚，完成239户市属企业改制，8.3万多名职工置换国企身份，209户企业实现资产重组。农村土地流转、水管体制改革逐步推进，集体林权制度改革完成主体任务。实施了新一轮政府机构改革，行政审批事项由1068项减少到328项。投融资体制改革成效显著，成功发行城投企业债券15亿元，整合组建了国资投资集团。科技、教育、文化、医疗和社会管理等领域改革全面推进。对外开放进一步扩大，开放型经济水平不断提高，五年累计引进国内资金980亿元，合同利用外资3.27亿美元，实现外贸进出口总额43.6亿美元。兰州被列为国家创新型试点城市、创业型试点城市和知识产权示范城市。

　　五是各项社会事业全面发展。着力推进科技创新，科技对经济的贡献率提高到55%。各级各类教育协调发展，校舍安全工程全面实施，教育质量水平进一步提高。“文化兰州”建设深入推进，建成了国学馆、秦腔博物馆、非遗陈列馆等一批特色文化设施。城乡医疗卫生条件不断改善，看病难、看病贵的问题逐步解决。“五城联创”取得积极成效，荣获全国创建文明城市工作先进城市称号。“双拥”工作深入开展,驻兰部队积极援建地方重点项目建设,为兰州经济社会发展做出了重要贡献。安全生产形势总体稳定，食品药品监管工作得到加强。圆满完成全国第六次人口普查工作。普法和依法治市工作被评为全国先进城市。民族团结、宗教和顺的局面进一步巩固。审计、统计、工商、质监、人口和计划生育、外事侨务、信访、防震减灾、残疾人救助、档案等工作取得了新的成绩。

　　六是人民生活水平较大提高。预计城市居民人均可支配收入和农民人均纯收入达到15890元和5229元，分别是2006年的1.7倍和1.8倍。五年累计用于民生的专项支出348亿元，年均增长19%。为民兴办实事89件，解决了一批群众关注的热点难点问题。城乡就业规模不断扩大，五年新增城镇就业人员25.6万人，输转农村富余劳动力132万人次。全面推行城镇居民基本医疗、新型农村合作医疗和基本养老保险等社会保障制度，各类保障标准逐年提高，覆盖城乡的养老、医疗、低保等保障体系基本建立。住房保障力度加大，城镇居民人均居住面积达到15平方米，比2006年增加1.2平方米。居民消费价格指数基本稳定，保证了广大群众的正常生活。

　　七是政府自身建设得到加强。自觉接受人大依法监督和政协民主监督，五年共办理人大代表建议、意见和政协提案4753件。完善了政府决策程序和议事制度，制定和修订地方性法规8部、政府规章50件。深入开展行政效能建设，规范工作制度、工作流程、工作行为、办公秩序、协调机制和内部监督程序，行政效率和服务水平明显提高。加强政务公开和督查工作，推进重大决策、重点工作和重点项目向社会公开承诺，提高了政府工作的透明度。启动“数字兰州”建设，强化网络问政和网上办公，形成了政府与市民良性沟通的机制。认真落实廉政建设责任制，各级政府工作人员廉洁从政的自觉性进一步增强，形成了志存高远、脚踏实地、认真工作、简单生活的良好氛围。

　　今年是本届政府的最后一年，也是“十二五”规划的开局之年。我们按照市委“再造兰州”战略和率先跨越发展的目标要求，把精力和财力向拓空间、调结构、强基础、惠民生等重点工作倾斜，全力以赴抓发展，统筹兼顾破难题，千方百计保民生，经济社会发展取得新的成效。预计全市生产总值增长15%，规模以上工业增加值增长15%，固定资产投资增长54%，社会消费品零售总额增长17%，地区性财政收入和地方财政总收入分别增长17%和18%，引进到位资金增长70%，城市居民人均可支配收入和农民人均纯收入分别增长13%和14%以上，其中生产总值、固定资产投资、引进到位资金和城乡居民收入增幅均创历史最好水平。

　　（一）全力推进“三区”发展，“3+8”板块经济体系逐步形成。一是兰州新区规划建设全面展开。编制完成总体规划、核心区控制性详细规划以及道路、生态等专项规划。开工建设10条核心区主干道路和兰秦快速通道，供水、供电、供热、供气、排水、垃圾处理等基础设施全面启动。舟曲灾后安置、行政服务中心、“千塘百湖”等重点项目加快实施。产业集聚效应初步显现，引进了中国铁建高端装备制造基地、三一重工、信源软件信息产业园等重大产业项目。生态建设同步实施，完成造林绿化1.6万亩，建成公共绿地120公顷。全年可完成固定资产投资160亿元，实现生产总值60亿元。二是两个国家级开发区增容扩区加快实施。高新区围绕建设一流科技新城、打造创新发展高地，优化“一区多园”规划布局，雁滩园区总部经济集聚效应进一步增强，七里河园区引进大学科技园、留学生创业园等项目，榆中园区基本完成规划编制工作。预计可实现生产总值190亿元。经济区改革管理体制和运行机制，设立安宁、西固、红古三个二级园区，签约引进奔驰产品支持中心、汇拓国际大酒店、兰海物流园等重大项目，中石油西部管道局总部、建材家居博览城等项目加快建设。预计可实现生产总值82亿元。三是县区产业园区竞相发展格局初步形成。城关九州开发区加大土地开发整理，引进光电与光伏产业园、沙地绿产研发基地等项目。西固物流新城着力发展现代物流产业，编制完成发展战略规划和专项规划。连海经济开发区红古园区清真明胶、油页岩炼油、特种石墨、高炉碳砖项目加快推进。连海经济开发区永登园区重点实施了腾达铁合金余热发电、大唐连城电厂节能技改等项目。榆中和平工业园区突出新材料产业发展，金川镍氢电池等项目加快建设。皋兰三川口工业集中区引进兰鑫钢铁高速线材、农产品加工配送中心等项目。

　　（二）加快发展培育多元支柱产业，项目建设取得突破性进展。一是健全完善产业发展推进机制。加强政府与企业、科研院校、金融机构的统筹协调，初步建立了“一个行业、一批主体企业、一批重点项目、一个科研机构、一个融资平台”的产业发展推进体系。二是产业招商全面展开。紧紧围绕多元支柱产业发展方向，细化40个方向64条产业链，凝炼500多个项目，完善了多元支柱产业招商项目库。强化产业工作组招商、以商招商和小分队招商，在北京、陕西、浙江、上海、湖南等地举办了大型招商推介活动，签约引进了一批重大项目，全年引进到位资金可达405亿元。兰洽会签约项目资金到位率和开工率分别达到42%和35%，是历届最高的一年。三是对接央企步伐加快。与中国铝业、国电集团、航天科技、中核集团等18家央企签约项目47个，其中14个项目开工建设，完成投资190亿元。佛慈制药首发上市获得证监会批准。完善“出城入园”政策，积极支持中央和省属在兰工业企业向兰州新区拓展，一批企业搬迁改造项目加快实施。四是项目攻坚力度加大。全面推行重大项目建设“指挥部模式”，集中开展“项目推进攻坚月”和“下基层、抓落实、破难题、促发展”活动，100项市列重大项目进展顺利，可完成投资330亿元。五是争取项目成效明显。91个项目列入国家规划，总投资1900亿元。争取中央和省上各类资金85亿元，比上年增长20%以上。特别是城市轨道交通全面完成前期工作，具备开工建设条件，西客站、南绕城高速等重大基础设施项目积极推进。

　　（三）高起点推进规划建设管理，城市功能形象大幅提升。一是城市规划不断完善。完成城市发展战略规划和综合交通规划研究，编制了第四轮城市总体规划，开展了城市地标景观、南北交通、停车场站、地下空间利用等专项规划修编工作。伏龙坪、白道坪和马滩地区整体开发积极推进。二是城市基础设施极大改善。完成七里河黄河大桥和中山铁桥维修改造，开工建设元通、金雁、深安黄河大桥。南山路绿色市场以东路段实现通车，北环路、西行线加快建设。针对城区道路破损严重、配套设施不全等问题，集中力量改造提升南北滨河路和老城区道路26条，完成路面热再生改造等244万平方米，整修人行道55公里，铺装道板砖28万平方米， 配套建设电力、通信、供热、供气、排水管网96公里，整治小街巷292条，改造公共绿地80公顷，较大改善了居民出行条件和生活环境。三是城市文化景观设施建设得到加强。完成白塔山、五泉山公园一期改造，免费向市民开放。建成兰州报业大厦、“八办”纪念馆新馆和彩陶馆。开工建设华夏人文始祖园、陇上名人园、城市雕塑群等主题公园。科技馆、体育中心、金城关创意文化产业园等项目积极推进。四是“畅交通”工程加快实施。制定了基于车联网技术的智能交通规划。启动交通控制、交通诱导、视频监控、非现场执法、快速公交线路控制等智能交通系统建设。实施了广场东口、雁滩什字、平沙落雁等交叉路口和重点路段的优化改造。建成人行天桥和地下通道10座、市区停车泊位2755个。五是环境污染治理力度加大。完成城区污水“全收集全处理”工程，雁儿湾污水处理厂建成使用，西固、盐场两座污水处理厂即将试运行。实施了市区81条洪道清淤疏浚。开工建设两座城市垃圾处理场。完成燃煤锅炉清洁能源改造2800蒸吨，实现热电联产和清洁能源供热5000万平方米。

　　（四）推进城乡一体化进程，农业和农村经济稳步发展。一是统筹城乡发展试点工作进展良好。坚持“中心带动、全域兰州”的理念，编制完成全市统筹城乡发展总体规划，基本实现小城镇和新农村建设规划全覆盖。加快体制机制创新，研究制定了林业生态可持续发展及城乡一体的社会保障、失地农民就业保障、土地管理和利用、金融保障、户籍管理、公共财政等政策措施。完成新农村示范点新建和续建项目23个。二是支农工作力度持续加大。公共财政投入继续向“三农”倾斜，全市农林水利支出14亿元，增长18.5%。加强对全膜双垄沟播技术的政策支持，完成播种面积50多万亩。加大农业项目扶持力度，明确产业链重点发展支撑项目，凝炼农产品招商项目25个，总投资14.6亿元。三是农业产业结构优化提升。特色农产品生产提档扩面，各类经济作物种植面积达到130万亩，高原夏菜产量突破200万吨，外销量达到105万吨。强化特色农产品包装推介和品牌创建，举办了高原夏菜产销对接会及“农业嘉年华”活动。四是农村基础设施建设进一步加强。完成三电、西电等4座泵站更新改造，新建水塘调蓄工程150座。实施农村安全饮水工程11项，受益农民10万人。建设农村公路650公里。建成农村沼气服务网点90个，新增沼气用户1300户。新建农村新住宅1万户，改造农村危旧房5000户。五是农村体制机制改革积极推进。建立了覆盖县乡村的土地流转平台，流转土地21.46万亩。启动了农村产权制度改革试点工作。集体林权制度改革通过省上验收。

　　（五）突出培育城市主题文化，成功举办一系列重大节会赛事。一是创造性打造节会赛事平台。注重发挥比较优势，积极经营城市，首次成功举办了兰州国际马拉松赛、环青海湖自行车赛兰州段比赛、中国MBA黄河漂流赛、中国机器人大赛暨RoboCup公开赛、首届中国车联网大会等大型节会赛事。二是区域性重大节会取得新成效。兰洽会办会水平明显提高，举办了系列主题论坛和专业展会，集中宣传推介了多元支柱产业及招商项目。首届“敦煌行·丝绸之路国际旅游节”成功举办，编排演出了文化旅游剧目《炫彩之旅》，增强了兰州旅游的吸引力。三是各种特色节会精彩纷呈。先后举办了春节文化庙会、合唱节、黄河风情文化周及动漫艺术节等系列节会活动。各县区文化旅游节会层次得到提高。这些节会赛事特别是兰州国际马拉松赛，场面规模之大、群众参与度之广、宣传报道力度之强、社会好评度之高，在兰州历史上前所未有，集中弘扬了黄河文化和城市精神，极大地提升了兰州的知名度和影响力，激发了全市人民的归属感和自豪感，增强了率先跨越的向心力和凝聚力，加快了“让兰州走向世界、让世界了解兰州”的步伐，对于宣传城市形象、优化投资环境、增添发展活力将产生持久而深远的影响。

　　（六）全力保障和改善民生，社会事业全面协调发展。一是为民兴办的实事进展良好。今年是为民兴办实事投资额度较大、涉及项目较多的一年，用于民生事业的支出可达114亿元，年初确定的十个方面26件实事到年底将全面完成，部分可超额完成。二是就业和社会保障力度加大。城镇新增就业5万人，登记失业率控制在4%。城市低保标准提高10%，农村低保标准由850元提高到1200元。新型农村社会养老保险试点工作深入推进，新增参保人数12.9万人。为低收入群众发放价格补贴3000多万元。开工建设保障性住房55557套，配租廉租房5000套，配售经济适用房3000套。新建商品房价格得到有效调控。三是城乡公共服务设施逐步完善。完成40所中小学校舍安全工程，21所城乡幼儿园加快建设，“两基”工作通过国家验收。开工建设省市妇女儿童活动中心。三县县医院、乡镇卫生院和村卫生所全部实现标准化。四是民生服务得到加强。继续推动解决房产证历史遗留问题，累计为近2万户城市居民登记发证。新建10个城区肉菜市场、100个农村便利超市。改造建设公厕150座，全市城市公厕实行免费开放。针对物价走势，积极采取临时价格干预措施，对主要蔬菜品种进行重点监管，有效平抑了物价上涨势头。

　　（七）创新和加强社会管理，全市社会和谐稳定。一是探索推广社会管理新机制。“民情流水线”得到中央和社会各界肯定，“虚拟养老院”服务模式在全国推广，“三维数字社区”在全市52个街道社区建成应用。构建省市联动信访工作机制，化积案、破难题，解决了一批复杂疑难的信访案件。二是“数字兰州”建设加快推进。建成政府门户网站群和全市政务内外网，以及协同办公、视频会议、智能会议、政务督查等系统，实现了政府重要会议无纸化、网络化召开。启动“市民卡”工程。市应急联动指挥中心启动建设，城关区“133”数字化工程投入运行。兰州被列为国家“两化融合”试点城市、地理空间信息试点城市和全国“无线城市”先行试点市。三是行政效能建设深入推进。在完善纵向流程的基础上，推进横向流程再造，实行了重点审批事项“网上收件、限时办结、上下互联、在线监督”。健全网络问政机制办法，办理人民网、政府网留言5000多条。通过市民见面会、现场办公、新闻发布会、手机短信等方式，认真倾听群众的意见建议，提高了政府工作的公信力。四是城市安全管理全面加强。社会治安综合治理不断加强，应急管理工作进一步改进。煤矿、交通、消防等领域安全生产状况明显改善，安全生产主要指标稳步下降。省市流通环节食品安全快速检测中心启动运行。建成10个食品药品安全示范乡镇街道、10条示范街、100家放心消费企业和门店。

　　各位代表，即将过去的五年，我们开创了全市改革发展的崭新局面，奠定了中心带动、率先跨越的坚实基础。这是在省委、省政府和市委的领导下，全市人民开拓创新、积极进取、奋力拼搏的结果，是市人大、市政协监督支持和社会各界共同努力的结果。在此，我代表市人民政府，向全市各族人民，向辛勤耕耘在各条战线的广大干部群众，向给予政府工作大力支持的各位代表、政协委员、各民主党派、工商联、人民团体和社会各界人士，向驻兰解放军指战员、武警官兵和公安民警，向离退休老同志致以崇高的敬意！向所有关心、支持和参与兰州现代化建设的同志们、朋友们、新闻媒体和广大网友们表示衷心的感谢！

　　五年发展的实践，进一步深化了我们对经济社会发展规律和市情的认识，积累了许多宝贵经验。我们深切体会到：兰州要实现经济社会全面协调可持续发展，必须牢固树立世界眼光和战略思维，咬定目标不放松，一张蓝图绘到底，在继承中创新，在创新中发展；必须立足发挥中心带动作用，同步推进老城改造和新区建设，拓展城市空间和完善城市功能；必须看到发展的后发优势，注重发挥比较优势，深入挖掘潜在优势，积极转化相对劣势，发展培育多元支柱产业，不断夯实经济发展的产业基础；必须始终坚持创新的理念，深化改革和扩大开放，激发内在活力，借助多重外力，以宽广的视野谋划发展、推动发展；必须善于经营城市，注重打造品牌赛事和特色节会，不断扩大城市影响力，提高城市带动力；必须把改善民生作为发展经济的根本出发点，倍加珍惜群众的意见，始终做到亲民、爱民、为民；必须把行政效能建设作为根本保障，加强制度建设和流程再造，提高政府的执行力、创新力、公信力和约束力。

　　五年发展的实践，我们也清醒地认识到，兰州经济社会发展仍然存在不少困难和问题，主要是：拓展城市空间的方向已经明确，但周期较长，任务繁重，形成新的城市结构布局还需要持续努力；统筹城乡发展取得较大成效，但城乡二元结构仍然突出，农民收入水平还比较低；经济发展速度不断加快，但对传统产业的依赖程度较高，多元支柱产业尚处于培育发展阶段；城市面貌有了明显变化，但基础设施功能薄弱，交通拥堵、大气污染等问题的解决是一个长期的过程；筹融资渠道逐步拓宽，但建设资金投入仍然不足，市场化融资的规模还不大；社会管理得到加强，但社会事业发展相对滞后，与广大群众日益增长的需求存在差距；行政效能有了一定提高，但部分干部观念缺乏创新，作风不够扎实，不能适应率先跨越发展的要求。我们一定要直面问题，正视不足，增强信心，振奋精神，求真务实，埋头苦干，努力把兰州经济社会发展不断推向跨越发展的新阶段。

　　二、今后五年政府工作的目标任务

　　今后五年，是我市实施“十二五”规划，加快推进“再造兰州”战略，实现率先跨越发展，全面建设小康社会的重要时期。刚刚闭幕的市第十二次党代会，是在这一新的形势下召开的一次承前启后、继往开来的重要会议。会议认真贯彻党的十七届六中全会精神，准确把握不断发展变化的市情，顺应全市人民创造美好生活的新期待，以“解放思想、创新实干、奋力推进兰州率先跨越发展”为主题，提出了今后五年全市发展的指导思想、奋斗目标和重点工作，这对于动员全市干部群众进一步解放思想、凝聚共识、开拓创新、加快发展，开创兰州各项工作的新局面，具有重大的推动作用和深远的影响。

　　按照市第十二次党代会的总体部署，政府工作将围绕率先跨越发展的总要求，立足提升城市影响力、发挥中心带动作用，以科学发展观为统领，以加快转变经济发展方式为主线，以改革创新为动力，以显著增加城乡居民收入为目标，深入实施“再造兰州”战略，加快推进兰州新区、高新区、经济区开发建设，加快发展培育多元支柱产业，加快基础设施建设步伐，加快城乡一体化进程，加快各项社会事业发展，努力实现经济社会发展水平的大幅提升，让全市人民生活得更加富裕、更加和谐、更加幸福。

　　（一）综合经济实力大幅提升。坚持“一产抓特色、二产抓延伸、三产抓转型、整体抓提升”，以发展培育多元支柱产业为重点，加快转变经济发展方式，在优化经济结构、强化节能减排的基础上实现“三个高于”、“七个翻番”。“三个高于”，即全市生产总值增速明显高于过去五年，高于全省平均水平，高于西部省会城市平均水平。“七个翻番”，即生产总值、固定资产投资、社会消费品零售总额、地区性财政收入、城市居民人均可支配收入、农民人均纯收入、引进国内外资金等主要经济指标，在2011年基础上翻一番，生产总值达到3000亿元以上。

　　（二）城市影响力和带动力大幅提升。实施第四轮城市总体规划和城乡统筹发展规划，坚持老城改造与新区建设相结合，推动城市结构大调整，城市功能大优化，城市环境大改善，全市城镇化率、城市规划道路建成率、绿化覆盖率和生活垃圾无害化处理率分别达到80%、80%、30%和75%。老城重在改造提升，全面抓好道路、轨道交通、黄河桥梁、公用设施、文体场馆和人居环境建设，加快小街巷、城中村、棚户区、国有企业危旧房、城乡结合部改造，城市综合服务功能不断完善，成为宜居宜业的美好家园。新区重在开发建设，强化基础设施，优化空间布局，突出产业带动，集聚效应明显增强，生态建设初见成效，与老城区的快速交通全面贯通，成为创新创业的现代新城。

　　（三）改革开放水平大幅提升。把改革开放作为加快发展的根本动力，全面深化各项改革，全方位扩大对内对外开放，大力发展非公有制经济，不断增强发展的生机活力。特别是抓住制约率先跨越发展的瓶颈，进一步通过改革破解难题，依靠创新增添优势，着力突破体制机制障碍。实施多元化开放战略，充分利用兰州新区等平台优势，积极承接东部地区产业转移，加大向西开放力度，加快兰白经济区建设，提高对外开放和区域合作水平，把兰州建成国家向西开放的重要平台。力争非公有制经济占生产总值的比重达到50%以上，引进到位资金达到1000亿元以上。

　　（四）文化软实力大幅提升。把文化建设摆在强市富民的全局位置，一手抓文化惠民，一手抓创意创作，综合发挥丝绸之路文化、黄河文化和民俗文化的比较优势，大力发展文化事业和文化产业，深化文化体制改革，推动文化产业成为全市最具活力的支柱产业。积极培育城市主题文化，建设一批标志性公共文化设施，打造具有兰州特色的文化精品力作，建设与经济发展和群众需求相适应的西部文化名城。全面加强精神文明建设和全国文明城市创建工作，推进公民道德建设工程，建立健全社会征信系统，促进公民素质和城市文明水平明显提高。

　　（五）城乡居民生活水平大幅提升。把改善民生作为工作的根本追求，坚持以人为本、统筹协调，加强和创新社会管理，推进科技、教育、卫生、体育等社会事业全面进步，促进人口与经济社会资源环境的协调可持续发展，逐步实现各项公共服务均等化。以创业带动和扩大就业，健全完善覆盖城乡的社会保障体系，进一步加快保障性住房建设，坚持每年为群众兴办实事，提高城乡居民收入水平和生活质量。深入推进“数字兰州”建设，加快信息化在生产生活领域的全面应用，着力提升城市交通智能化管理水平和畅通能力。进一步加强社会安全、生产安全、食品药品安全、环境安全和交通安全管理，以平安和谐的城市环境保障民生、服务民生、惠及民生。

　　三、2012年的政府工作

　　2012年是新一届政府的开局之年，兰州的发展步入一个新的阶段。我们将以“三区”建设为支撑点，以发展培育多元支柱产业为切入点，以破解发展难题为着力点，以提升城市影响力为突破点，以保障和改善民生为根本点，努力实现各项工作的新突破。全市经济社会发展的主要预期目标是：生产总值增长15%以上；工业增加值力争增长15%；全社会固定资产投资增长35%；社会消费品零售总额增长19%；地区性财政收入和地方财政总收入分别增长16%；城市居民人均可支配收入和农民人均纯收入分别增长14%；城镇登记失业率控制在4%以内；居民消费价格涨幅保持在国家和省上控制范围内；节能减排完成国家和省上下达的目标任务。

　　（一）夯实兰州新区发展基础，增强“三区”承载能力。把兰州新区建设和高新区、经济区增容扩区作为率先跨越发展的平台支撑，一手抓基础设施建设，一手抓项目引进和产业发展，加快构建以兰州新区为核心的“3+8”板块经济体系。

　　着力增强兰州新区产业集聚功能。健全完善路网框架体系，启动建设迎宾大道、北快速路等15条道路，“四纵四横”主干线全面竣工，兰秦快速通道水秦段通车。建成第二给水厂主体、1号热源厂和330千伏变电站，加快第一污水处理厂、生活垃圾填埋场等项目建设。配套实施学校、医院、文体场馆等公共服务设施。大力推进以经济林为主的生态建设，完成造林绿化3.5万亩。加大招商引资和产业项目建设力度，中国铁建高端装备制造基地、三一重工、大成绿色镀膜等20个重点项目建成投产。力争完成固定资产投资200亿元，实现生产总值100亿元。

　　加快高新区新一轮发展。优化提升雁滩产业孵化核心区，新建一批科技研发和企业孵化项目。增强七里河园区整体功能，启动星火机床、四方压力容器、嘉恒节能建材等重点产业项目。抓好榆中园区基础设施和配套工程建设，初步形成产业集聚效应。确保生产总值达到200亿元。

　　推进经济区西进扩区。健全完善二级园区配套设施。加快实施安宁园区荒山整理开发，综合开发土地1万亩，启动建设沙九产业园。强化西固园区统一规划和基础建设，培育和引进一批现代物流、新材料、精细化工项目。突出红古园区有色金属新材料产业发展，加快兰海物流园、防火保温材料基地等项目建设。确保生产总值达到100亿元。
